# PROphylactic closed incision Negative-PRESSure treatment in open incisional hernia repair: Protocol for a multicenter randomized trial (PROPRESS study)

**DOI:** 10.1016/j.conctc.2024.101256

**Published:** 2024-01-10

**Authors:** Mads Marckmann, Nadia A. Henriksen, Peter-Martin Krarup, Frederik Helgstrand, Peter Vester-Glowinski, Mette Willaume Christoffersen, Kristian Kiim Jensen

**Affiliations:** a Digestive Disease Center, Bispebjerg Hospital, Denmark; b Dept. of Gastrointestinal and Hepatic Diseases, Herlev Hospital, University of Copenhagen, Herlev, Denmark; c Department of Surgery, Zealand University Hospital, Koege, Denmark; d Department of Plastic Surgery and Burns Treatment, Rigshospitalet, Denmark; e Department of Surgery and Transplantation, Rigshospitalet, Copenhagen University Hospital, Denmark

**Keywords:** Incisional hernia, Open hernia repair, Surgical site infection, Closed incision negative pressure therapy (ciNPT), Quality-of-life

## Abstract

**Background:**

Negative Pressure Therapy in closed incisions (ciNPT) after surgery has shown positive effects including reduction of Surgical Site Infection (SSI) incidence. In patients undergoing elective open incisional hernia repair, however, ciNPT is not standard care, perhaps due to high-quality evidence still not provided. This study hypothesizes that this patient group would benefit from ciNPT by reducing wound complications and improving postoperative quality of life.

**Method:**

This is a multicenter Randomized Controlled Trial (RCT) including a total of 110 patients allocated in a 1:1 ratio with one intervention arm and one active control arm receiving ciNPT (i.e., Prevena™) and standard wound dressing, respectively. The primary outcome is the incidence of SSI at 30 days postoperatively and secondary outcomes are 1) pooled incidence of Surgical Site Occurrence (SSO), 2) patient-reported pain and satisfaction with the scar, and 3) hernia-related quality of life.

**Conclusion:**

Patients undergoing elective open incisional hernia repair are fragile with a high risk of wound complication development. This multicenter RCT seeks to deliver the high-quality evidence needed to establish the role ciNPT must play for exactly this group with the aim of reducing SSI incidence and health economic costs, and finally improving quality of life. There are no theoretical or clinical experience of unwanted consequences of this treatment.

## 1 Introduction

### 1.1 Background and objectives

In the recent years, prophylactic Negative Pressure Therapy has been utilized for treatment of complicated wounds within orthopedic and vascular surgery, resulting in reduced risk of surgical site infection, wound dehiscence and length of stay [1,2]. A reduced risk of surgical site infection has also been shown after caesarean section [3]. There are only a few small studies on the possible benefit of prophylactic negative pressure therapy for closed incisions after clean-contaminated abdominal operations [[4], [5], [6], [7]].

The development of an incisional hernia is associated with a poor wound healing potential, therefore patients undergoing incisional hernia repair by definition have impaired wound healing (i.e. the hernia) often due to comorbidities including diabetes, obesity and a history of smoking making them at risk of wound complications. The incidence of surgical site infection after incisional hernia repair is high, ranging from 18.7 to 66.0 % [8,9]. Wound complications are independently associated with prolonged hospital stay, re-operations, re-admissions, mortality and an increased health- and socioeconomic burden [[10], [11], [12]]. Patient-reported quality of life is reduced in patients with an incisional hernia and besides better healing potential closed incision negative pressure therapy may also improve the cosmetic of the scar which may affect patients’ body image [13,14]. Thus, prophylactic measures to reduce this risk of wound complications and improve quality of life in patients undergoing incisional hernia repair are needed.

This study hypothesizes that closed incision negative pressure therapy (Prevena™, KCI/Acelity, San Antonia, TX, USA) compared with a standard wound dressing reduces the incidence of surgical site infections after open repair of incisional hernia.

## 2 Methods trial design

This is a multicenter randomized double-blind, controlled trial comparing an intervention (closed incision negative pressure therapy) with a standard treatment, respectively. The aim is to randomize 110 patients in a 1:1 allocation ratio with 55 patients in each arm. There have been no significant changes to methods after trial commencement. This protocol article was conducted respecting the CONSORT guidelines when applicable, including an expected flow chart diagram (Fig. S1) [15].

### 2.1 Participants

Patients are recruited from three centers in Denmark: (Digestive Disease Center at Bispebjerg Hospital in Copenhagen, Department of Gastrointestinal and Hepatic Diseases at Herlev Hospital, and Surgical department of Zealand University Hospital Koege). Patients are included in the study during the planning of surgery in the outpatient clinic or within a week before surgery at the planned visit in the department of surgery. Patients are pragmatically presented for the study by either operating surgeons, nurses in the out-patient clinic, or the study's PI at the time of their last outpatient visit before surgery, which is usually 1–4 weeks before scheduled surgery. Formal and final inclusion is eventually conducted by the study's PI approximately one week before surgery at which point the participants also provide their informed consent. Eligibility criteria for patients are shown in Table 1.

**Table 1:** Inclusion criteria and exclusion criteria for participants of the PROPRESS-study.

Inclusion criteria	Exclusion criteria
Age ≥18 years	Active superficial or deep wound infection
Patients undergoing elective open incisional hernia repair	Inability to provide informed consent/inability to understand the language of the informed consent and/or questionnaires
	Allergy to silver

### 2.2 Interventions

For the intervention group, the following procedure is utilized: After skin closure, Prevena™ is applied using the following technique: the sterile foam dressing from the package is placed on the entire length of the incision on top of which a sterile dressing is placed, ensuring air tightness. The tube for the interface pad is connected and a continuous negative pressure of 125 mmHg is applied. The Prevena™ system is removed on the morning of postoperative day 3. This is reasoned by the fact that the patients are usually discharged on this day, but also because the Prevena™ is designed for application for a minimum of 2 days, and up to 7 days, why 3 days will ensure a minimum of 48 h application accounting for variation of surgery duration. In the unexpected case of the dressing mandating removal or changing before day 3, there are two scenarios: 1) the event implies one of the study's outcomes and is treated by standard guidelines or 2) the event is of minor character such as technical device error or blood or serous fluid, in case of which the device and/or dressing is replaced by a new one.

For the control group, after skin closure, a standard operative dressing, i.e., **evercare**®Adhesive surgical dressing, OneMed, is applied in the entire length of the incision. This operative dressing is removed in the morning of postoperative day 2 reflecting the usual clinical practice and standard nurse care procedures in the hospitals involved. As for the intervention group, only in the case of blood or serous fluid mandating changing of the dressing, will this be done prior to postoperative day 2.

All patients will receive standard preoperative skin preparation with 0.5 % chlorhexidine and preoperative antibiotic prophylaxis, i.e. a solution of 4 g piperacillin/0,5 g tazobactam or 1500 mg cefuroxime for patients with penicillin allergy, administered within 30 min prior to the incision and repeated after 3 h, if surgery is still ongoing. In case of allergy towards both piperacilline/tazobactam and cefuroxime, other prophylactic antibiotic is administered. Body temperature is monitored by the anesthesiologist team in the operating room.

The costs of the wound dressings are covered by either the hospital's budget or the study's funding grant with no co-payment required for participating patients.

### 2.3 Outcomes

The primary outcome of the study is the incidence of surgical site infection within 30 days postoperatively. Surgical site infection is diagnosed as defined by Center for Disease Control [16]. Secondary outcomes include: 1) pooled incidence of surgical site occurrence within 30 days, 2) patient-reported pain and satisfaction with the scar and 3) hernia-related quality of life. Approximately one week before date of surgery, patients are initially presented with the questionnaire regarding quality of life either by phone or an outpatient visit. Subsequently, all outcome variables are assessed 30 days post-operatively by a phone interview conducted by the study's primary investigator (PI), whom is a physician not performing any of the study's surgeries. Surgical site occurrence is defined as either surgical site infection, wound hematoma, skin necrosis, seroma or superficial wound dehiscence. Patient-reported outcome effects will be assessed by validated questionnaires, i.e. the POSAS v2 (the Patient and Observer Scar Assessment Scale) and the EuraHS^1^ Quality of Life questionnaire. Patient baseline characteristics and postoperative outcomes will be compared to evaluate potential statistical differences. Table 2 lists all selected variables collected peri-operatively.

**Table 2:** Registered data, variables, and outcomes.

Preoperative baseline
Age [nominal: number]
Sex [categorical]
Male, Female
Number of previous abdominal operations [nominal: number]
Number of previous incisional hernia repairs [nominal: number]
Smoking status [categorical]
Active smoker, Non-smoker
Body Mass Index [kg/m2]
Chronic obstructive pulmonary disease [categorical]
Yes, No
Diabetes [categorical]
Yes, No
Chronic Kidney Disease (CKD) class [categorical]
1, 2, 3, 4, 5
American Society of Anesthesiology (ASA) class [categorical]
1, 2, 3, 4, 5
Site of incisional hernia [categorical]
Midline, Upper left, Lower left, Upper right, Lower right, Other
Orientation of hernia incision [categorical]
Horizontal, Vertical
Anticoagulative medication [categorical]
Anticoagulants, Platelet aggregation inhibitors, Acetylsalicylic acid, None
Serum albumin level [numerical: g/L]
Botox [categorical]
Yes, No

Intraoperative
Type of procedure/repair [categorical]
Rives-Stoppa (RS), RS + Transversus Abdominis Release (TAR), onlay, Intraperitoneal Onlay Mesh repair (IPOM), RS + Endoscopic Component Separation (ECS), other
Operative time [numerical: minutes]
Length of incision [numerical: centimeters]
Length of suture used for fascial closure [numerical: minutes]
Contamination grade [categorical]
clean, clean-contaminated, contaminated, dirty
Type of mesh [Categorical: Name]
Mesh dimensions horizontal and vertical [Numerical: centimeters]
Placement of subcutaneous drain [categorical]
Yes, No
Maximal horizontal fascial defect from CT scan [numerical: centimeters]
Maximal vertical fascial defect from CT scan [numerical: centimeters]
Commentary option (e.g. perioperative complications)

Postoperative – follow-up
Surgical site infection (SSI) within 30 days [categorical]
Yes, No
Surgical site occurrence within 30 days [categorical]
Surgical site infection, Wound hematoma, Skin necrosis, Superficial wound dehiscence, Seroma
Patient-reported pain and satisfaction with the scar at 30-day follow-up [POSAS v. 2 questionnaire]
Hernia-related quality of life [numerical: euraHS]

### 2.4 Sample size

The reported incidence of surgical site infections after incisional hernia repair vary to a great extent. The sample size calculation is based on a retrospective study by Soares et al. including 199 patients undergoing open incisional hernia repair [17]. Previously published data from the current trial's main inclusion site show patients similar to those from the study by Soares et al. (mean body mass index <35 kg/m2, American Society of Anesthesiologists' score II-III, and all but one patient with a wound contamination grade of clean or clean-contaminated) [18]. Soares et al. reported that the incidence of surgical site infection in patients treated with standard dressing was 32 % compared to 9 % in patients treated with negative pressure wound therapy. Thus, considering a significance level of 0.05 and a statistical power of 80 %, a total of 94 patients are required for randomization with 47 in each group. Thus, taking dropouts into account the plan to include 110 patients, yielding 55 in each arm. No interim analysis is planned as this was not required by the relevant authorities.

## 3 Randomization

Randomization was performed using computer-generated sequences of varying block sizes. This was done by www.sealedenvelope.com and the randomization list was implemented in the electronical research database RedCap by the PI of the study.

### 3.1 Blinding

When patients are in the operating room and the surgeons are performing skin closure, a non-sterile person in the operating room will call the primary investigator, who then via RedCap presses the “Randomize” button, and by phone gives the answer of standard or intervention treatment. Thus, until this point, everyone, including patients, surgeons, and study conductor is blinded to intervention assignment. The 30-day registration of both primary and secondary outcomes are then assessed by the study's PI who was blinded to the treatment allocation. Patients are not blinded after treatment allocation.

### 3.2 Statistical methods

Continuous measures will be reported as mean (standard deviation) and Student's t-test will be applied unless data are not normally distributed. Categorical parameters will be compared across groups by the chi-square test. *P*-values below 0.05 are considered statistically significant.

## 4 Results

### 4.1 Participant flow and recruitment

Inclusion period started March 1st, 2023, and will continue until the total number of 110 patients has been reached, which expectedly will take between 1 and 1½ years, based on the average planned numbers of trial-relevant operations in the centers involved. Thus, inclusion period is expected to end in the Spring or Summer of 2024, and follow-up for this protocol will eventually end 30 days after the last included patient.

### 4.2 Baseline data

Please refer to Table 2, which includes all pre-operative baseline demographic and clinical characteristics that will be collected for all included participants. With respect to sex and gender-based analyses (SGBA), this study uses the definition of sex as a binary category retrieved from the participants’ social security number at the time of inclusion.

### 4.3 Patient and public involvement

The research questions were developed with the patient's perspective as highly important in accordance with the State-of-the-Art requirements for modern research involving Patient Related Outcome Measures (PROMs) [19,20]. This is a patient-centered study, since the aim is to improve patient reported outcomes. However, no patients were directly involved in the study design.

### 4.4 Ethics, harms, monitoring, and trial registration

This trial seeks to reduce the incidence of wound complications in patients undergoing open incisional hernia repair. There is limited theoretical and no clinical experience with unwanted side effects of closed incision negative pressure therapy. Allergies to any device-related parts, as listed in Table 1, may – as in any other setting – be unknown, thus, debut of patch-allergy when exposed to Prevena™ cannot be pre-excluded, in which case the treatment will be interrupted. Some patients may be bothered by device-related alarm noises or the fact that the pump must stay close to the patient at all times, however, these cannot be categorized as serious harms. Hence, over all it is hypothesized that the intervention will be an improvement compared with the standard treatment, without increasing the risk for severe side-effects. This includes both a reduction in wound complications and better patient-reported quality of life. Thus, the expected benefits from participation outweigh potential drawbacks and is therefore considered ethically appropriate. Institutional ethics approval was received for the study (Danish Medical Research Ethics Committee #2115253) and informed consent was obtained for experimentation with human subjects. Lastly, the trial conforms to the Helsinki Declaration, which is a statement of ethical principles for medical research involving human subjects, including research on identifiable human material and data [21].

## 5 Discussion

The PROPRESS study seeks to provide the possible role of closed incision negative pressure therapy in patients undergoing incisional hernia repair. Patients with incisional hernia have a compromised wound healing potential and often comorbidities such as obesity, diabetes and are active smokers, which makes them at risk for wound-related complications. Minimal invasive surgery has offered a reduced risk of surgical site infection compared to open approaches, however, there is still a need for interventions to further reduce this risk in open surgery settings [22].

Studies on the use of closed incision negative pressure therapy showed a reduction in surgical site infection, wound dehiscence and length-of-stay in orthopedic, abdominal, colorectal, and obstetric surgery [2]. A meta-analysis including five randomized controlled trials in patients undergoing open abdominal surgery with primarily closed laparotomy incisions found no difference in surgical site infection, however, there was a statistically significant heterogeneity across studies, which involved all from pancreaticoduodenectomy to general laparotomy [7]. The same meta-analysis involved different NPT-devices and reported non-standardized alternative measures to reduce surgical site infection (e.g., antibiotics). A second large meta-analysis involving more than 25 interventional trials included studies from a wide variety of surgical specialties and concluded that the evidence was of low or very low certainty for all outcomes including surgical site infection [23]. Finally, a third meta-analysis [24] including five studies focusing solely on open ventral hernia repair did conclude that closed incision negative pressure therapy significantly reduced the incidence of surgical site occurrence and surgical site infection compared to standard dressings, however, only one randomized trial by Bueno-Lledó et al. was involved [25], which carried several methodological limitations: 1) it was single-center-based and 2) included only patients with midline incisions with horizontal defects >4 cm, and 3) excluded patients with initial emergency hernia repair, all factors limiting generalizability. Furthermore, 4) they used a NPT-device demanding in-hospital-stay for 7 days, which poorly adheres to the rapidly emerging enhanced recovery protocols that also applies to this present study, where patients per standard are discharged on post-operative day 3. Lastly, Bueno-Lledó et al. 5) did not evaluate any patient-reported outcomes measures, as this present study is designed to do. Thus, there is still a need for studies to explore the possible benefit of closed incision negative pressure therapy for closed abdominal incisions after incisional or primary ventral hernia repair with the aim to reduce the risk of surgical site infection as it is known that surgical site infection lead to a significant increase in morbidity and mortality, hernia recurrences, prolonged hospital stay, and increased hospital costs [26]. Also, this study proposes that closed incision negative pressure therapy indirectly improves patients’ quality of life, supported by other studies that showed improvement in quality of scarring when using closed incision negative pressure therapy compared to standard care [13].

The study design of a randomized, double-blind multicenter trial, minimizes selection bias including convenience sampling, which was present in the majority of the previous studies on this subject, which were retrospective in design, a factor also limiting the real-life assessment on patient-reported quality of life [8]. A retrospective analysis including 199 patients that were operated over a 5-year-period showed that there were missing variables of interest possibly confounding the results, such as hernia defect size and number of previous surgeries. In the same study all procedures were carried out by a single surgeon, which compromises external variability and ignores the possible effect of surgeon's expertise improvement over a 5-year period [17].

As the costs of the negative pressure therapy-device (Prevena™) in the experimental arm of 55 patients are in the order of 11.000 euros in total equaling approximately 200 euros per patient, this makes studying the cost-benefit ratio of importance. This current trial will not assess this question initially, however, it is planned to perform a separate cost-benefit study, which is readily enabled by this study's basic design, but will require additional economic patient-specific data extracted from the involved centers' financial departments, which demands more project approvals and time. However, surgical site infections are associated with increased hospital stay and pose a substantial economic burden. Some studies report a doubling of costs for a patient that is diagnosed with a surgical site infection due to more medication use, higher risk of prolonged or additional length-of-stay in hospital, and a higher risk of hernia recurrence and re-operation [11,[27], [28], [29]]. Thus, it is unlikely to think that the cost of using a negative pressure therapy device should be less advantageous than the overall expected benefits.

Lastly, the power calculation for the current study was based on previous reports on the effect of negative-pressure therapy in patients undergoing incisional hernia repair. As in every trial there is a risk of a type-II error, however in that case the current study will still add valuable data to future a meta-analysis of randomized trials comparing negative-pressure therapy to conventional wound dressing.

## Generalizability

The surgical procedure for incisional hernia repair varies. To achieve a high degree of external validity three factors should be highlighted: 1) all types of open incisional hernia repair will be allowed in the current study, 2) three different centers are involved in the trial, and 3) different hernia specialists will be performing the operations. This study does not include emergency hernia repairs, mainly because emergency incisional repairs are rare events, and thus implies a limitation for extrapolation of results.

## Funding statement

This work was supported by 10.13039/501100009708Novo Nordisk Foundation Project Grants in Surgical Research 2020 (grant #0065841).

## Category of submitted manuscript

Protocol article for a randomized clinical trial.

## Is the paper based on a previous communication to a society or meeting

No.

## Originality

This article is an original work, has not been published before, and is not being considered for publication elsewhere in its final form, in either printed or electronic media.

Any republication of the data (e.g. in secondary analysis) will not constitute redundant publication, will not breach copyright, and will reference the original publication.

## Authors’ contributions

KKJ is responsible for the conception of the work. MM drafted the article. NH, FH, MW, PVG, MM and KKJ all contributed equally with critical revision of the article.

## Trial registration

MREC (#2115253). Clinicaltrials. gov (NCT05050786).

## CRediT authorship contribution statement

**Mads Marckmann:** Project administration, Writing – original draft, Writing – review & editing. **Nadia A. Henriksen:** Investigation, Project administration, Supervision, Writing – review & editing. **Peter-Martin Krarup:** Supervision, Writing – review & editing. **Frederik Helgstrand:** Writing – review & editing. **Peter Vester-Glowinski:** Writing – review & editing. **Mette Willaume Christoffersen:** Investigation, Project administration, Writing – review & editing. **Kristian Kiim Jensen:** Conceptualization, Funding acquisition, Investigation, Methodology, Project administration, Supervision, Visualization, Writing – review & editing.

## Declaration of competing interest

The authors declare that they have no known competing financial interests or personal relationships that could have appeared to influence the work reported in this paper.

## Data Availability

Data will be made available on request.

## Appendix A Supplementary data

Fig. S1 Expected flow chart for the Propress Trial Fig. S1
